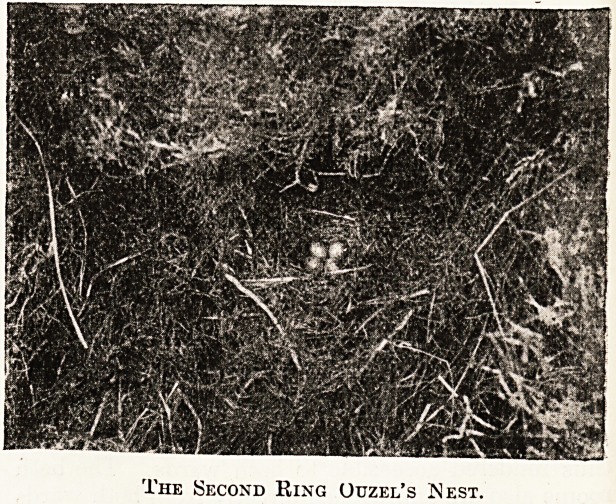# The Haunt of the Ring Ouzel
*The first article of this series appeared last week.


**Published:** 1913-04-19

**Authors:** 


### RELAXATIONS AND HOBBIES.

### II.—The Haunt of the Ring Ouzel.*

It was our last day on the moor, and we turned back towards camp with profound regret. But our luck, having found us late, declined to desert us at once. On the way up we had marked one of the

The Meadow Pipit's Nest.

meadow pipit's nests which abound everywhere on the moor as being particularly suitable for photography. The photograph was to be taken on the way home, but, approaching the spot from a different direction, we failed to pick up our marks of identification. A long and tedious search followed which was eventually attended with success and led to a further discovery. I was casting round to pick up my original line of approach when, turning suddenly under the influence of that sort of extra sense which a field naturalist must develop, I saw "out of the tail of my eye" a ring ouzel disappearing over a bank near me which I had passed and, I think, searched on the way up. I could not say for certain where the bird had come from, but there was about its flight that self-conscious air of anxiety to avoid observation, which is characteristic of a nesting bird; so I made a mental note of the spot and resumed my search for the meadow pipit's nest. This I had marked by taking bearings between certain bushes and rocks which I now shortly found. The meadow pipit's nest was successfully photographed, and we proceeded to search, for the ring-ouzel's nest. I walked almost directly to it—a beautiful nest with four eggs in it, and so placed that it could easily be photographed. A photograph was secured without delay, and we trudged back to camp with the comfortable feeling that after all our short expedition had ended gloriously.

### The Song of the Ring Ouzel.

It is perhaps worth while to record that during the whole of our brief sojourn on the moor I never once heard the song of the ring ouzel so as to be certain of it beyond a doubt. Once or twice I heard a hint of the song in the distance, but if it was the ring ouzel's song at all it was not sustained, and I could never follow it up. It has been seen that we found both our ring ouzels' nests by flushing the female bird from the nest more or less unexpectedly; the male bird was not *en évidence* at all. In previous years and at earlier seasons I had seen, as far as I can recollect, only male birds, and had been attracted to them by the song. Similarly in early spring on any part of the moor one may see innumerable male meadow pipits rising in the air with shrill lark-like notes, then fluttering to earth again, with tails upturned and wings outspread, uttering those soft sweet notes which this bird shares with the tree pipit. But in May, though we heard the alarm call of the meadow pipit all round us every day, and were constantly chancing upon their nests, I do not think we heard the song of the male at all.

### The Silent Moor.

This (with regard to the meadow pipit) is an afterthought, but the sights and sounds of those days of holiday live in my memory, and, looking back, I am impelled to note this difference between the birds of the moor and those others of our meadows and thickets who (the males) are so eloquent during the period of incubation. On

The Second Ring Ouzel's Nest.

the high grounds, in May, no song was to be heard, and only occasional sounds—the bleating of a startled sheep, the cry of a vigilant curlew, or the fussy alarm-note of the meadow pipit—encroached upon the pervading silence of the moor.

* The first article of this series appeared last week.

## Figures and Tables

**Figure f1:**
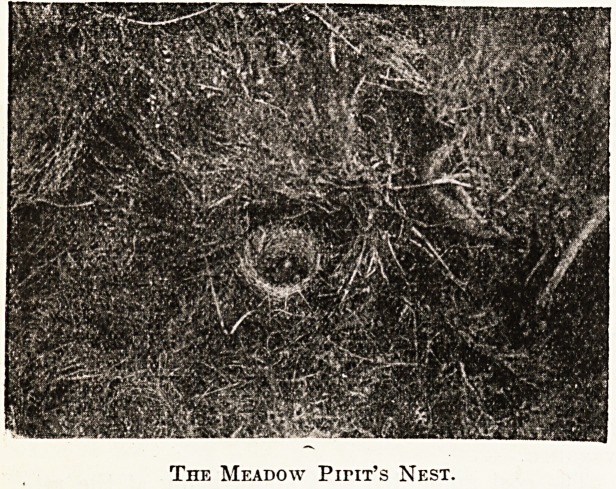


**Figure f2:**